# A fluidic platform for mobility evaluation of zebrafish with gene deficiency

**DOI:** 10.3389/fnmol.2023.1114928

**Published:** 2023-04-06

**Authors:** Xiaoyu Jia, Yibo Feng, Wenju Ma, Wei Zhao, Yanan Liu, Guangyin Jing, Jing Tian, Tao Yang, Ce Zhang

**Affiliations:** ^1^State Key Laboratory of Photon-Technology in Western China Energy, Institute of Photonics and Photon-Technology, Northwest University, Shaanxi, Xi'an, China; ^2^School of Physics, Northwest University, Shaanxi, Xi'an, China; ^3^Shaanxi Key Laboratory for Theoretical Physics Frontiers, Institute of Modern Physics, Northwest University, Shaanxi, Xi'an, China; ^4^Key Laboratory of Resource Biology and Biotechnology in Western China, Ministry of Education, School of Medicine, Northwest University, Shaanxi, Xi'an, China

**Keywords:** active fluidic, fluidic device, zebrafish mobility, gene deficiency, mechanical vibration

## Abstract

**Introduction:**

Zebrafish is a suitable animal model for molecular genetic tests and drug discovery due to its characteristics including optical transparency, genetic manipulability, genetic similarity to humans, and cost-effectiveness. Mobility of the zebrafish reflects pathological conditions leading to brain disorders, disrupted motor functions, and sensitivity to environmental challenges. However, it remains technologically challenging to quantitively assess zebrafish's mobility in a flowing environment and simultaneously monitor cellular behavior *in vivo*.

**Methods:**

We herein developed a facile fluidic device using mechanical vibration to controllably generate various flow patterns in a droplet housing single zebrafish, which mimics its dynamically flowing habitats.

**Results:**

We observe that in the four recirculating flow patterns, there are two equilibrium stagnation positions for zebrafish constrained in the droplet, i.e., the “source” with the outward flow and the “sink” with the inward flow. Wild-type zebrafish, whose mobility remains intact, tend to swim against the flow and fight to stay at the source point. A slight deviation from streamline leads to an increased torque pushing the zebrafish further away, whereas zebrafish with motor neuron dysfunction caused by lipin-1 deficiency are forced to stay in the “sink,” where both their head and tail align with the flow direction. Deviation angle from the source point can, therefore, be used to quantify the mobility of zebrafish under flowing environmental conditions. Moreover, in a droplet of comparable size, single zebrafish can be effectively restrained for high-resolution imaging.

**Conclusion:**

Using the proposed methodology, zebrafish mobility reflecting pathological symptoms can be quantitively investigated and directly linked to cellular behavior *in vivo*.

## Introduction

Zebrafish (*Danio rerio*) is a small freshwater teleost, carrying features, such as the genome, brain patterning, and the structure and function of neural and physiological systems, similar to other vertebrate species (Lockwood et al., 2004; Lieschke and Currie, 2007; Kily et al., 2008; Strähle et al., 2012). For example, studies with known mammalian neurotoxic and cardiotoxic agents have shown that these substances caused similar effects in zebrafish (Ton et al., 2006; Kari et al., 2007). Therefore, zebrafish have been widely employed in neuro-pharmacological studies (Kokel et al., 2010; Ellis and Soanes, 2012). To date, the behavior of the zebrafish has been well described and believed to represent brain functions including sensory, motor, and cognitive behavior (Gerlai, 2003; Miklosi and Andrew, 2006; Parng, 2006). However, it remains challenging to investigate zebrafish behavior under dynamic environmental conditions and at the cellular level.

To explore the locomotion and the cellular activities behind zebrafish, it is crucial to maintain dynamic environmental conditions during high-resolution fluorescence imaging. Field studies revealed that zebrafish live in secondary and tertiary channels connected to a mainstream, where the flow rate is in the range of 3.5–13.9 cm/s (Arunachalam et al., 2013). In 2016, Suriyampola et al. found that zebrafish become more aggressive and form less cohesive groups when they were transitioned from still water to a water environment of low flow rate (Sykes et al., 2018). However, in most studies, the behavior of zebrafish larvae was studied using the touch-evoked approach, in which a stick was used to manually poke the target zebrafish (Lu et al., 2021). For studies at the single cell level, e.g., the development of early neurons, zebrafish has to be anesthetized, which lacks dynamic stimuli like the continuous flow in the wild (Park et al., 2000; Palaisa and Granato, 2007; Santanu et al., 2015). Therefore, a flow field manipulation platform, on which live zebrafish are isolated and stabilized in a flowing current, is highly demanded.

Active fluidic devices, which can manipulate liquid samples at high precision, have been employed in zebrafish behavioral studies for various functional purposes including entrapment, transportation, culturing, and perfusion. For example, a microfluidic bioreactor with an aperture structure was designed to immobilize zebrafish embryos for local site-specific stimulation (Shen et al., 2009). Son et al. reported a two-plate droplet-based “digital” microfluidic technology for the on-chip transporting of zebrafish embryos *via* an electrowetting-on-dielectric (EWOD)-mediated electromechanical force (Son and Garrell, 2009), but none of these approaches allow the entrapment of individual zebrafish in dynamic environmental conditions, e.g., continuous flow, for high-resolution fluorescence imaging.

To address these issues, we designed a droplet-based fluidic device, where zebrafish are confined in a droplet to suppress long-distance migration. The flow pattern surrounding single zebrafish (i.e., one, two, and four recirculating flow patterns) was adjusted by tuning the vibration amplitude and frequency of the glass substrate underneath the droplet. It is uncovered that in a droplet with four recirculating flows, the mobilities of zebrafish can be evaluated by assessing the angle of zebrafish with respect to the inward flow direction (instead of long-distance migration), i.e., wild-type zebrafish tend to swim against the flow, and the others with disrupted mobility are forced to stay at a balanced position. Moreover, zebrafish remain immobile in the droplet for a relatively long period of time, i.e., 1–2 s for the wild-type zebrafish and more than 3 s for the ones with disrupted mobility, which is considerably longer than the required exposure time. We, therefore, conclude that the proposed strategy is suitable for studies of zebrafish behavior in a continuous flow and simultaneously allows high-resolution fluorescence imaging.

## Experimental section

### Device fabrication and materials used

The fluidic device is composed of a glass slide spin-coated by a thin polydimethylsiloxane (PDMS) layer, a PDMS reservoir for the droplet, and a holder for the electric motor (M20, DC1.5V ~ 3V) (Supplementary Figure S1A). Both the PDMS reservoir and holder are produced by casting PDMS on the top of the 3D-printed molds (CR-3040S, CREALITY, China; minimum printing line width of 0.4 mm and height of 0.1 mm) (Supplementary Figures S1B, C). In detail, to produce molds with a smooth surface, a printing material named PolysmoothTM (Polymaker, China) was used (Feng et al., 2020). The printed template was then positioned amid 75% ethanol or isopropyl alcohol for polishing, the duration of which depends on the structural features of the templates (30 min to 1 h). Following the standard protocol of PDMS casting, the mixer of the PDMS base and the curing agent at a ratio of 10:1 was poured into the 3D-printed molds, degassed, and baked at 45°C for at least 12 h for solidification.

Those PDMS parts are then bonded to the PDMS-coated glass slide after plasma treatment (Supplementary Figure S1D). The 3D-printed lever carrying two metal springs (i.e., wire diameter of 0.25 mm, outer diameter of 2.4 mm, and length of 9 mm) is connected to the electric motor. The elastic deformation of the spring while knocking on the glass slides ensures continuous rotation and thus controlled knocking frequency and amplitude. During the experiment, the whole device is mounted on the top of the microscope stage, where only four corners of the glass slide are fixed. Repeated knocking of the metal spring on the glass slide causes vibration, which propagates across the slides and reaches the droplet. Consequently, mechanically caused deformation of the droplet leads to variations in the internal flow patterns (Zeng et al., 2022).

### Zebrafish culture

All experimental procedures on zebrafish were approved by the Experimental Animal Management and Ethics Committees of Northwest University and carried out in accordance with the approved guidelines (NWU-AWC-20190617Z). Adult zebrafish of AB wild-type (wt) strain and Tg (elavl3:eGFP) transgenic line were raised with a standard light cycle (14 h light, 10 h dark) (Kim et al., 1996; Lu et al., 2020). Embryos were obtained by natural crosses and staged as described earlier (Mwaffo et al., 2017). Fertilized embryos were cultured in the E3 medium (NaCl: 0.29 g/L, KCl: 0.012 g/L, CaCl_2_: 0.036 g/L, MgSO_4_: 0.04 g, pH 7.4) at 28.5°C. For experiments in the vibrating droplet, all zebrafish were tested 72 h post-fertilization (hpf). Among them, wild-type fish were incubated without any treatment (Type I). The zebrafish with disrupted motility (Type II) was treated with 0.012% concentration of anesthetic [3-aminobenzoic acid ethyl ester methanesulfonate, SIGMA (A5040)] for 3 ~ 5 min to simulate the zebrafish with damaged motor nerves. For the recovery group (Type III), the anesthetized fish was put into the water and then waited for 5 min before tests.

### Image acquisition and data analysis

A time-lapse of zebrafish within the miniature swimming pool, which is 12 mm in diameter and 1.5 mm in height, was recorded using a commonplace mobile camera at a frame rate of 20 fps. The videos were then extracted and processed using ImageJ software for better visualization and contrast. Trajectories of zebrafish were obtained by determining the fish centroids and tracking their movement using a customized Matlab program. The mean square displacement (MSD) of the zebrafish is calculated using the formula: MSD(τ) = < Δr(τ)^2^> = < [r(t+τ)-r(t)]^2^>, where r (t) is the position of the zebrafish centroid at time t, τ is the lag time between the two positions to calculate the displacement Δ r (τ) = r (t + τ) – r (t), and the average < ...> designates a time-average over t.

To assess zebrafish mobility in the flow field generated by the percussion of a droplet, the PDMS-based fluidic device was placed on a Nikon TiE inverted fluorescence microscope. The flow field at different substrate vibrating amplitudes and frequencies was examined by tracking the movement of fluorescent particles (polystyrene microspheres) at a frame rate of 30 fps, the trajectories of which were then analyzed using the software LabPIV. The macroscopic behavior (e.g., rotation) was imaged using 4 × objective. The capacity of the device in high-resolution fluorescent imaging was demonstrated using a 40 × objective. In all experiments, the temperature was maintained at 28.5 and 100% RH using a stage-top incubator.

## Results and discussion

### Design and operation of the fluidic device

We, herein, attempt to evaluate the mobility of different zebrafish groups, e.g., the ones with lipin-1 deficiency and anesthetized ones (Lu et al., 2021). Optimally, both the macroscopic fish movement and its neuron system at the single-cell level are monitored in real-time to establish direct connections. Even with the state-of-art motor stage and automated tracking program (Pardo-Martin et al., 2010; Choudhury et al., 2012), it is difficult to catch up with the so-called burst-and-coast swimming style of zebrafish and take fluorescent images in dynamic environmental conditions.

**Figure 1:**
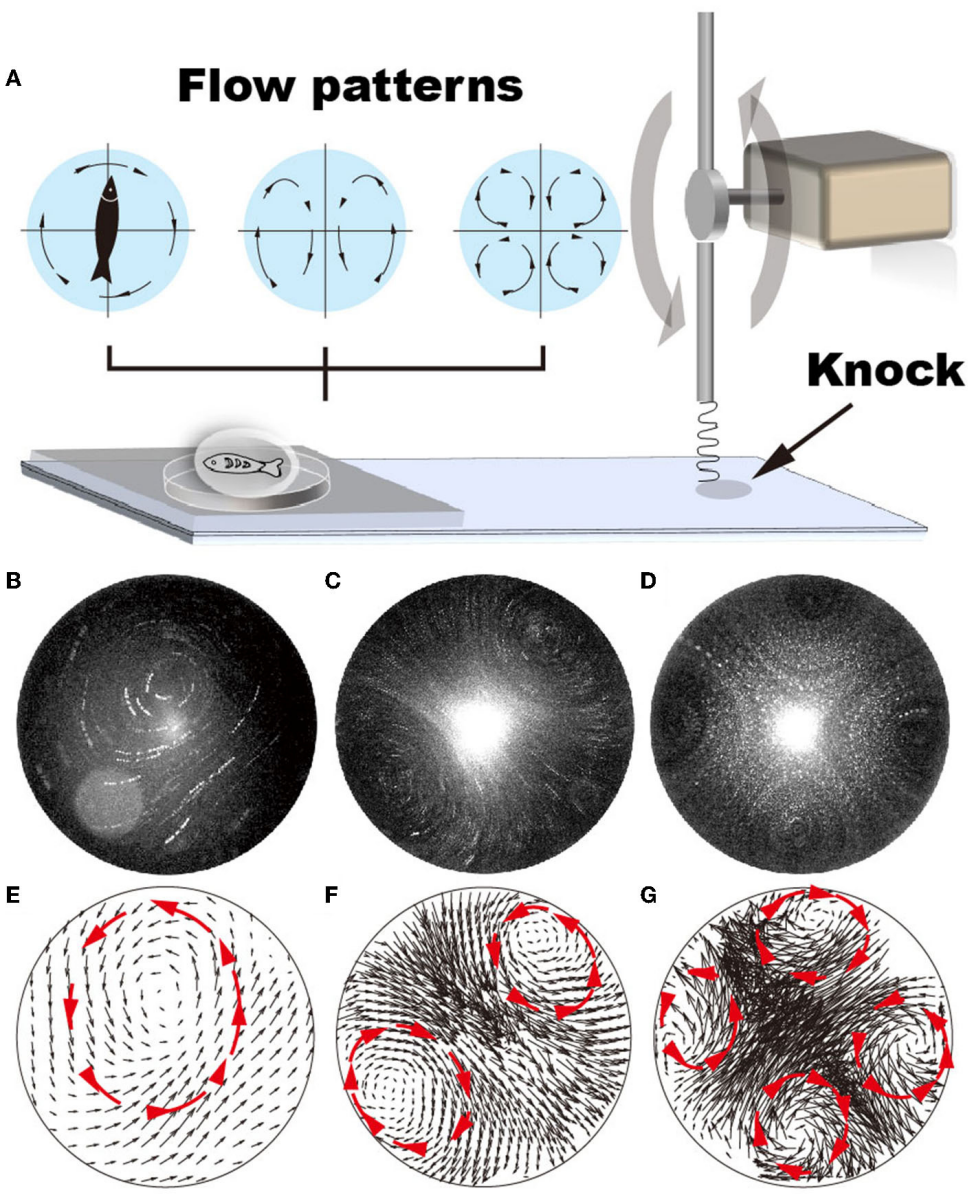
Various flow patterns can be generated by adjusting the vibration frequency of the glass substrate, i.e., from 22 to 52 Hz. **(A)** Schematic shows that the vibrating frequency is regulated by repeatedly knocking the glass substrate. **(B–G)** Using GFP fluorescent particles (1 μm in diameter) as a tracker, flow patterns generated by vibrating the glass substrate at vibrating frequencies ranging from 22 to 52 Hz are plotted. **(A, D)** Single recirculating flow zone generated at 22–35 Hz frequency; **(B, E)** two recirculating flow zones generated at 36–47 Hz frequencies; **(C, F)** four recirculating flow zones generated at 48–53 Hz frequencies.

To meet the challenges, we developed a simple-to-use fluidic device, which was produced using 3D printing (Figure 1A and Supplementary Figure S1). In brief, the device is composed of a glass slide and a small electric motor (M20, DC1.5V~3V), which is mounted on a glass slide *via* a holder made by PDMS. The electric motor is connected to two levers carrying a metal spring. Rotation of the levers drives the metal springs to repeatedly knock on the glass substrate and causes vibration, which propagates to the droplet housing zebrafish. The actuation amplitude and frequency of substrate vibration are controlled by adjusting the spring length and electric power, respectively (Supplementary Video S1). To ensure that the droplet is sitting on a substrate with controllable stiffness and hydrophobicity, a PDMS reservoir is placed on the top of the glass slide. The droplet shape can then be adjusted by tuning the hydrophobicity of the substrate (i.e., the contact angle) *via* polyvinyl alcohol processing following plasma treatment (Trantidou et al., 2017). To prevent unwanted evaporation, the droplet fluidic device was positioned in a sealed box at 100% RH, and a temperature of 28.5°C to maintain a healthy environment for zebrafish (Zeng et al., 2022).

Using fluorescent particles of 1 μm in diameter as a tracker, our results demonstrate that the various flow patterns can be generated in the droplet by gradually changing the droplet size (*via* liquid volume), shape (*via* hydrophobicity of the glass substrate), the vibration amplitude (*via* lever length), and frequency (*via* motor rotation). When the vibration amplitude is large (i.e., > 0.2 mm underneath the droplet), the droplet easily deforms, resulting in an uncontrollable and unstable flow pattern (Supplementary Videos S2, S3). It is difficult to determine the frequencies at which flow patterns with two and four recirculating zones were generated (Supplementary Figures S2E–H, M–P). In contrast, at low vibration amplitude (i.e., < 0.1 mm), the flow pattern shows dependency on the vibration frequency (Supplementary Figures S2A–D, I–L). For example, when the droplet volume is 10 μL and is positioned under a vibration amplitude of 0.1 mm, the flow pattern gradually transits from random to four recirculating flow patterns with increasing vibration frequencies, i.e., random → one recirculating zone → four recirculating → two recirculating → random (Figures 1B–D and Supplementary Figure S2). The transitioning process varies with changing droplet shape, determined by liquid volume and substrate hydrophobicity. Notably, the velocity distributes ununiformly in the droplet (Figures 1E–G), showing considerably lower velocity at the center of each recirculating zone.

No regular flow pattern was generated when vibration is induced to bulk solution in a 96-well plate (Supplementary Video S4). These results indicate that the vibration of the substrate induces droplet deformation, which subsequently causes internal flow. The distinctive conformational transition of the droplet at various experimental conditions (i.e., the droplet size, shape, vibration amplitude, and frequency) leads to various internal flow patterns. Consistently, studies by Yang et al. reveal that shape deformation of the droplet leads to variations in the internal flow patterns (Yang et al., 2019). In contrast, the hydrophobicity of the substrate plays no crucial role in regulating the flow pattern in the droplet (Supplementary Figure S2).

### Mechanical forces on constrained zebrafish under various flow patterns

**Figure 2:**
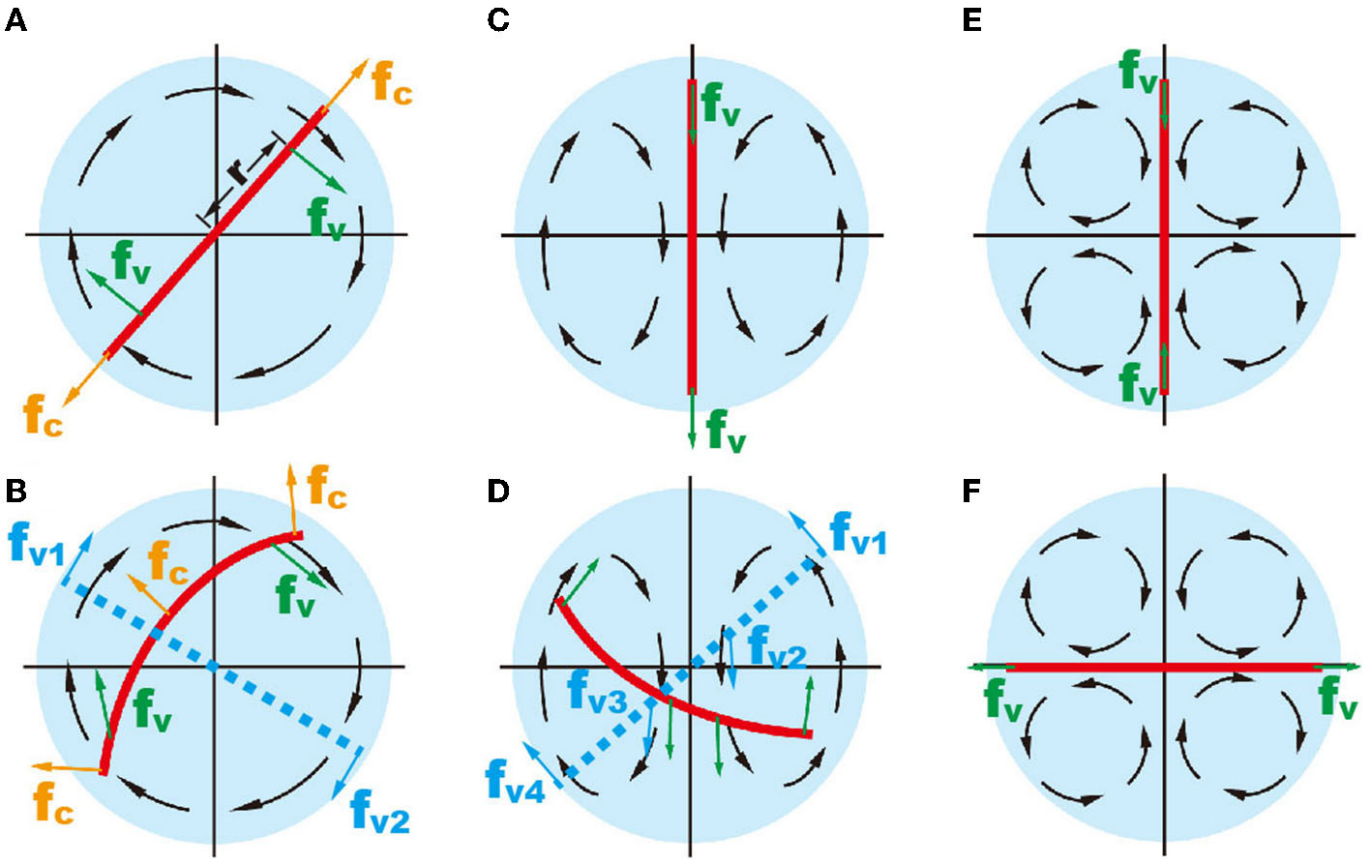
Schematic showing the mechanical forces on an inextensible object (red line) in different flow patterns. **(A, B)** The object in one recirculating flow zone. **(C, D)** two recirculating flow; and **(E, F)** four recirculating flow. In **(B, D)**, the red solid lines represent a deformable object and the blue dashed lines represent an unbendable object.

Assuming that a zebrafish is an inextensible object within the droplet, the mechanical forces, which force the object to move, include gravitational forces pulling zebrafish down to the bottom, viscous force *f*_*v*_ forcing zebrafish to go with the flow, and centrifugal forces *f*_*c*_ causing zebrafish to bend (Figure 2). When droplet dimension is comparable to zebrafish, the movement in longitude direction is trivial, and therefore, the effects of gravitational force are neglected. In a droplet with a single recirculating flow, viscous force *f*_*v*_ at both ends provides torque forcing the object to rotate with the flow (Figure 2A). When the object rotates about its own central point and is unbendable, the torque of centrifugal forces *f*_*c*_is zero. Considering also the fact that the flow velocity is considerably lower in the central region of the recirculating flow (Figure 1E), the total torque can, then, be simplified as *Torque* = *f*_*v*1_·*r*+*f*_*v*2_ *nbvcxb*·*r* = 2·*f*_*v*_·*r*. Deformation (i.e., bending) of zebrafish in the flowing environment causes deviation of zebrafish from the droplet center (Figure 2B), leading to unbalanced centrifugal forces and driving the object to the marginal region, where *f*_*v*_ is maximized (Supplementary Video S5) (Ha and Gary Leal, 2001).

In two recirculating flows, the torque on zebrafish is zero at the balance position (Figure 2C). Active zebrafish movement leads to increased torque, i.e., $Torque=\;f_{v1}\cdot r-f_{v2}\cdot r^{{}^{\prime }}+f_{v3}\cdot r^{{}^{\prime }}-f_{v4}\cdot r\;=\;\left(f_{v1}-f_{v4}\right)\cdot r+\left(f_{v2}-f_{v3}\right)\cdot r^{{}^{\prime }}$, where *r* and *r*′ denote the actuation points of viscous forces of two recirculating flow patterns (Figure 2D). Obviously, the action of one recirculating flow pattern (i.e., *f*_*v*1_ and *f*_*v*2_) provides counterforce for another one (i.e., *f*_*v*3_ and *f*_*v*4_). Therefore, forces on the zebrafish in the droplet are mostly balanced. The viscous force in two recirculating flow patterns causes zebrafish to bend.

**Figure 3:**
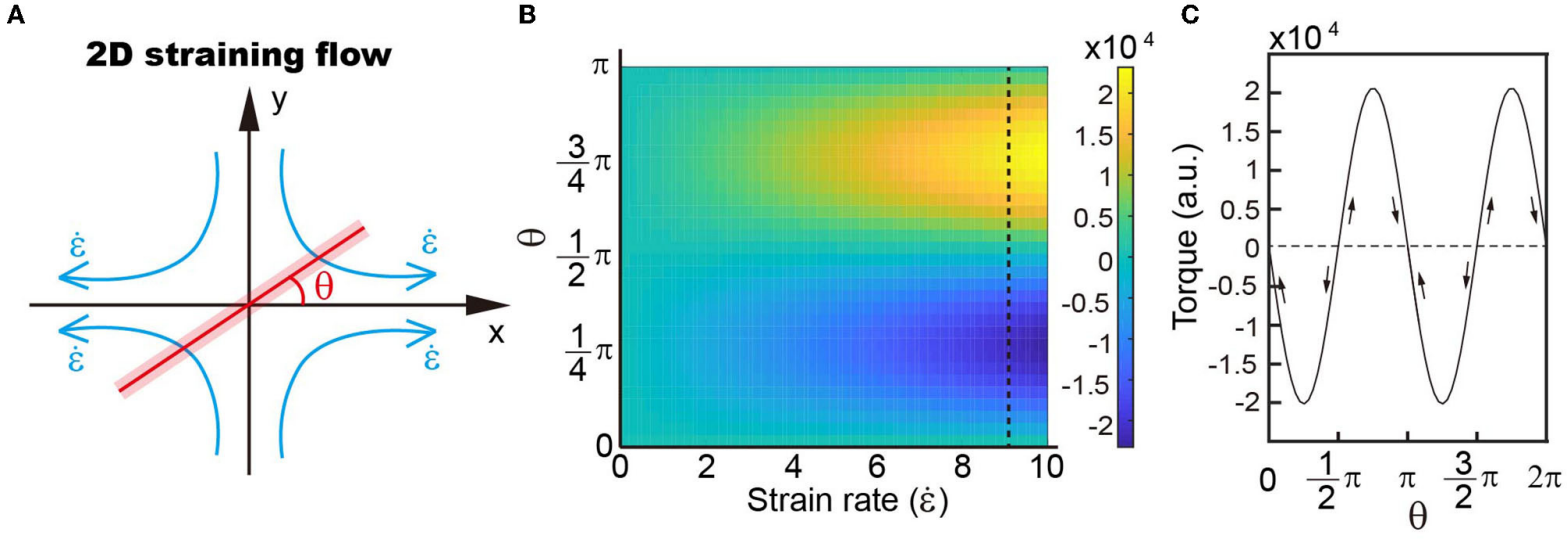
**(A)** Schematic showing the simplified model of four recirculating flow patterns. **(B)** Distribution of torques with different strain rates and object angles with respect to the outwards flow shown in a. Our results demonstrate that the torque distribution depends on the angle rather than the strain rate. **(C)** Variations in torque when the object rotates within the velocity field.

In the droplet with four recirculating flow zones, there are two balanced positions, where the total viscous torques are zero (Figures 2E, F). To illustrate, we simplify the flow pattern to a 2D straining flow $\left(u,v\right)=\left(\overset{•}{\varepsilon }x,-\overset{•}{\varepsilon }y\right)$, where $\overset{•}{\varepsilon }$ is the strain rate of the straining flow simplified from the velocity field within the droplet (Figure 3A). The flow field can be written in polar coordinates as follows:


$$\left(\frac{u_{r}}{u_{\theta }}\right)=\left[\begin{array}{c} cos\theta \;sin\theta \\ -sin\theta \;cos\theta \end{array}\right]\left(\frac{u}{v}\right),\;$$

where *u*_*r*_ is the radial velocity; *u*_θ_ is the circular velocity; *u* is the velocity in the *x* direction; and *v* is the velocity along the *y* axis. The parallel and perpendicular drag coefficients of a slender object, with length *L* and cross-section radius *a*, are *c*_||_ = *4*πμ*/c* and *c*_⊥_ = *8*πμ*/c*, respectively, where μ is the liquid viscosity and *c* = *2ln(L/a)-1* is the drag coefficient correction (Batchelor, 1970). The relative velocity of the object can then be written as $u_{r}^{{}^{\prime }}=u_{r}$, and $u_{\theta }^{{}^{\prime }}=u_{\theta }-\overset{•}{\theta }\cdot s$, where $\overset{•}{\theta }=\frac{d\theta }{dt}$ is the radial velocity of the object and *s* is the arc length. When the object is positioned at angle θ, the torque is then


$$\begin{array}{l} Torque=\int s\cdot c_{⊥}\;u_{\theta }^{{}^{\prime }}ds=-\frac{8\pi \mu \overset{•}{\varepsilon }}{c}\frac{L^{3}}{3}\sin\left(2\theta \right).\; \end{array}$$

It is demonstrated that the orientation of torque varies with the rotation angle of zebrafish in a small droplet, i.e., with a size comparable to zebrafish. There are two balanced positions, where the torque on zebrafish is zero, i.e., position-1: π/2 and position-2: π (Figure 3B). Deviation of the object from both position-1 (π/2) and position-2 (π) leads to increased torque (Figure 3C). The difference is that direction of the torque is the same as zebrafish rotation at position-1, forcing the object to go further way, which is indicated by the arrows in Figure 3C. At position-2, the torque direction is opposite to zebrafish movement and thus drives the object to return to its original position.

### Evaluation of zebrafish mobility within the droplet

**Figure 4:**
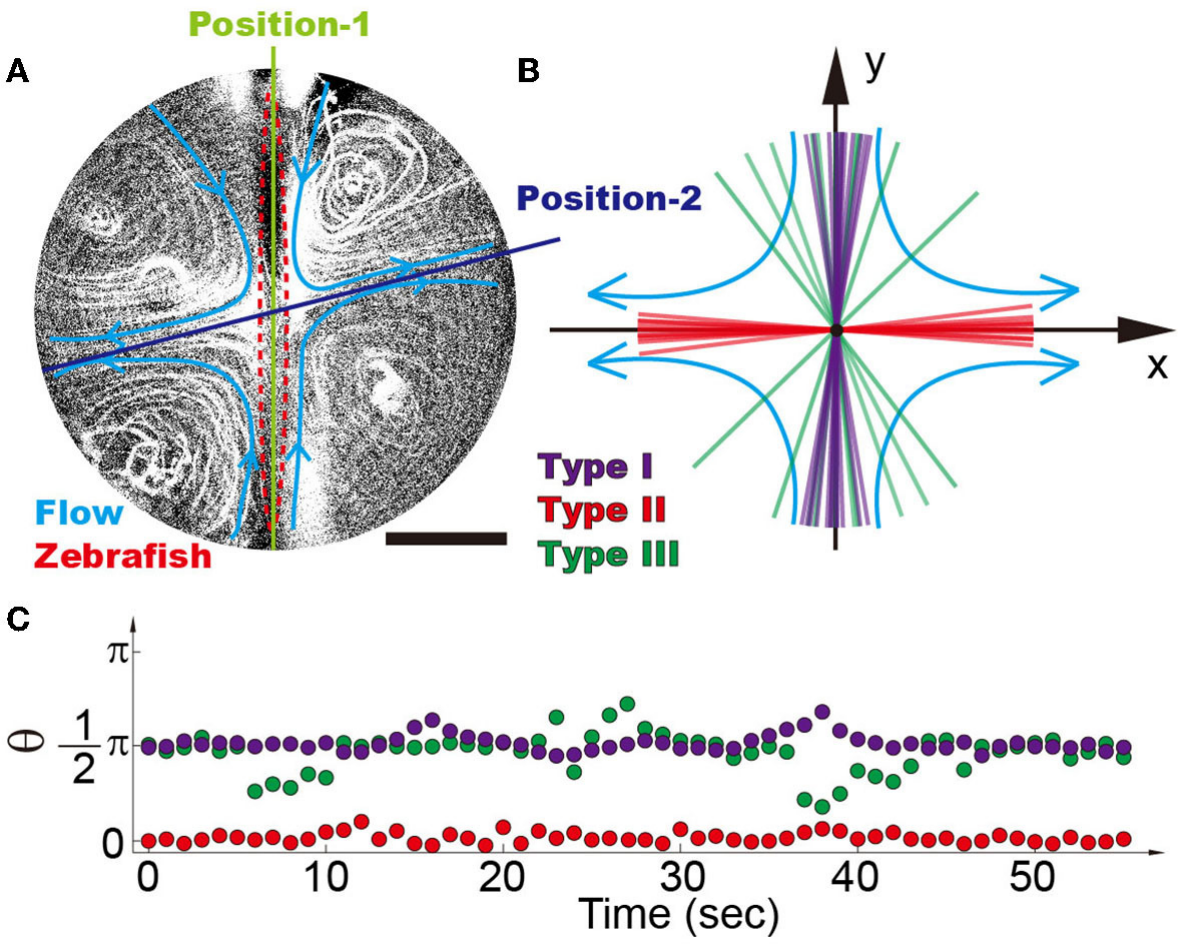
**(A)** Four recirculating flow zones were generated surrounding the zebrafish. It is demonstrated that there are two balanced positions, i.e., position-1 denotes the inward flow and position-2 denotes the outward flow. **(B)** Position distribution of different types of zebrafish shows that high-mobility fishes can be easily distinguished from the low-mobility group, i.e., type I (purple), type II (red), and type III (green) zebrafish. Each line reflects the average angle of individual zebrafish over a time span of 5 min. **(C)** Traces of single zebrafish's movement indicate that different types of zebrafish can be distinguished from each other. Each dot represents the position angle of a zebrafish at different time points. The scale bar denotes 1 mm.

By generating four recirculating flow patterns surrounding a zebrafish (Figure 4A), we observed that zebrafish with high mobility tend to stay at position-1, where the heads point against the flow direction (i.e., π/2 in Figure 4B) (Chu et al., 2004), which is consistent with the previous observation that wild-type zebrafish tend to swim against the current (Mwaffo et al., 2017; Oteiza et al., 2017). The narrow region, where wild-type zebrafish (type I) stabilize themselves within the time span of 5 min, ranges approximately from 80 to 100. As indicated in the previous section, the deviation of zebrafish from position-1 induces a torque forcing the zebrafish to move toward position-2. Therefore, the stabilization of the zebrafish at position-1, where the head of the zebrafish is against the current, suggests good mobility, balance, and stability (Figure 4A) (Mwaffo et al., 2017; Oteiza et al., 2017). In contrast, the zebrafish with disrupted mobility (type II) is forced to position-2 (angle of ~0±10°) (Supplementary Video S6), suggesting the loss of mobility to escape from the mechanical trap (Figures 3C, 4B, C).

These results demonstrate that zebrafish with intact mobility can distinguish themselves from disabled ones. Unlike the zebrafish in the miniature swimming pool (Supplementary Figure S3), whose responses depend on the zebrafish state and may sometimes require poking with a stick, the ones in droplets are constantly exposed to environmental stimulation of continuous flow. Wild-type zebrafish have to be active in order to stay at position-1, which eliminates the possibility of false results. For example, wild-type Fish04 in Supplementary Video S7, by lacking stimulation, remains immobile for a relatively long period of time as compared to others. No similar phenomena were observed in the vibrating droplet. Even though the recovered fishes position themselves in a relatively broader region than the wild-type fishes (~90 ± 40°), these fishes can be easily distinguished from disabled ones (type II). The positioning of different fishes is stable within the duration of experiments (Figure 3C), indicating that the proposed device is a suitable platform for assessing zebrafish mobility.

**Figure 5:**
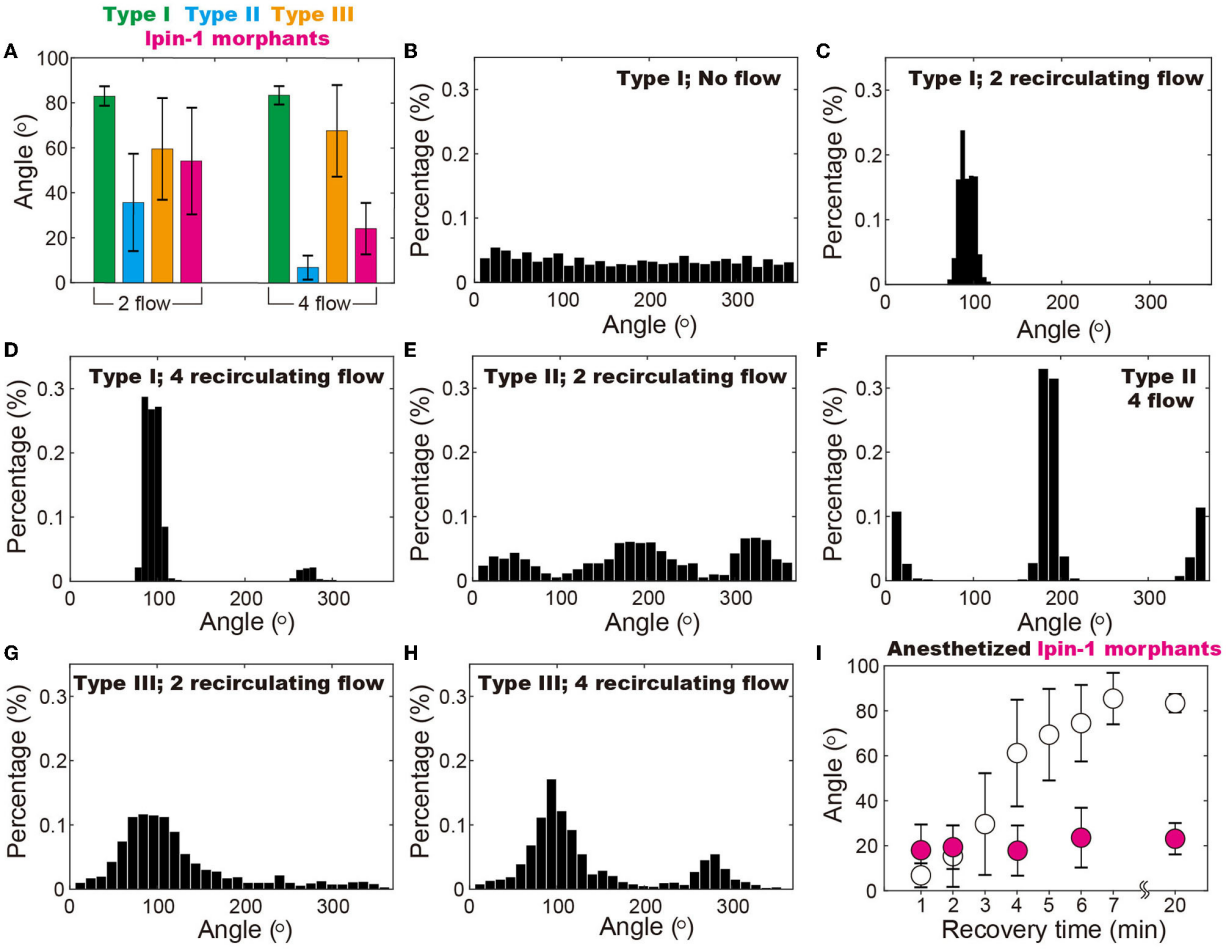
Position of zebrafish in the vibrating droplet. **(A)** Positions of different types of zebrafish indicate that in the droplet with four recirculating flow zones, zebrafish with disrupted mobility (i.e., type II and the ones with lipin-1 deficiency) can be better distinguished from the wide type ones (i.e., type I) as compared to the two recirculating flow conditions. **(B–H)** Position (i.e., angle) distribution of different types of zebrafish in various flow patterns including two and four recirculating flow patterns. **(I)** In the four recirculating flow patterns, anesthetized zebrafish gradually regains mobility and moves from 0° to 90° within a time span of 20 min. In contrast, zebrafish with lipin-1 deficiency remain immobile throughout the experiments. In **(B)**, each zebrafish was maintained in the droplet for 20 min at 100% RH and 28.5°C to ensure that the random movement of zebrafish is recorded. Movements of 5–10 zebrafish are individually monitored in droplets with various flow patterns and also more than 50 zebrafish in droplets with no flow.

The conclusion is further supported by statistical analysis of 5–10 zebrafish under each experimental condition (Supplementary Section 1). It is demonstrated that the anesthetized zebrafish (i.e., type II with compromised mobility) and the ones with motor dysfunction (i.e., lipin-1 deficiency) can be better distinguished from the wild-type zebrafish (type I) in the four recirculating flow patterns as compared to the two recirculating flow patterns (Figure 5A). In the two recirculating flow patterns, all five type I (i.e., wild-type) zebrafish stay in a narrow region of ~30 (Figure 5C), where their heads point against the current (Figures 5A, C, D). The probability can be calculated using $p={\left(\frac{30}{360}\right)}^{5}\cdot C_{12}^{1}$, which gives a *p*-value of 4.8 × 10^−5^. In the four recirculating flow patterns, the head of four type I zebrafish points to both 90 and one toward 270 (Figure 5D), all of which fluctuate in a range of ~30. This gives a *p*-value of ~0.01. Even though zebrafish with disrupted mobility (type II and lpin-1 morphants) position their heads at different angles ranging from −20 to 20 and 180 to 220 in four recirculating flow patterns, the *p*-value remains as small as 0.024, which is smaller than the threshold for statistical significance 0.05. In contrast, zebrafish recovering from anesthetization show a broad distribution of angles in the vibrating droplet (Figures 5G, H). A clear dependence of zebrafish position distribution on the recovery time is observed, indicating that the relative angle of zebrafish is closely associated with its mobility (Figure 5I).

### Fluorescence imaging of zebrafish in a vibrating droplet

**Figure 6:**
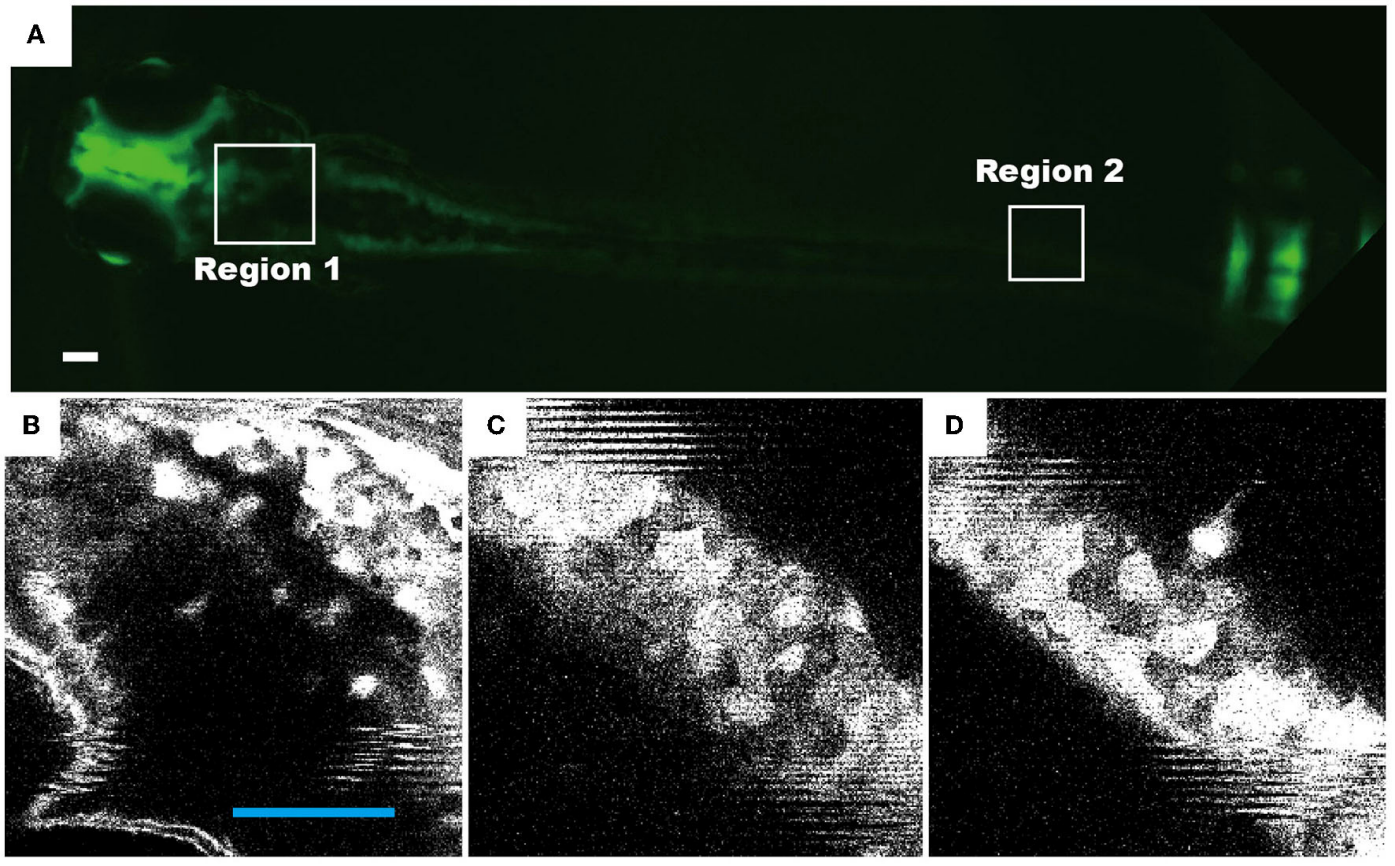
Representative fluorescence images of live zebrafish, which were transfected and maintained in a droplet of four recirculating flow patterns. The neuronal cells of transgenic zebrafish permanently express the HuC-GFP minigene (10.7 kb). Fluorescence images of 72 hpf living zebrafish: **(A)** as a whole fish, **(B)** the head region [region 1 as indicated in **(A)**], and **(C, D)** the tail region (region 2). Scale bars in all figures denote 100 μm.

Another key feature of the device is that zebrafish are maintained in a droplet of comparable size, where the movement of zebrafish is restricted. The stabilization of zebrafish in the four recirculating flow patterns allows us to study zebrafish behavior in dynamic environmental conditions during high-resolution fluorescence imaging. Our results demonstrate that using 4 × objective, the distribution of neurons (Figure 6A) and movement of internal organs (Supplementary Video S8) can be clearly imaged at an exposure time of ~50 ms. At higher magnification, i.e., 40 × , neurons and their axons were observed (Figures 6B–D), showing a similar resolution and clarity as those obtained from the fixed samples. We, therefore, conclude that the droplet-based fluidic device provides opportunities for us to quantitatively investigate the behavior of zebrafish larvae and perform high-resolution fluorescence imaging.

## Conclusion

In this study, we presented a fluidic device produced using 3D printing and soft lithography. By inducing vibration on the underneath glass substrate, flow patterns including one, two, and four recirculating flow patterns were generated in a confined space. Taking advantage of the fact that zebrafish tend to swim upstream (i.e., against the flow direction), which depends greatly on its mobility, we demonstrate that the mobility of the zebrafish is proportional to its angle in the four recirculating flow patterns. Therefore, the fluidic device can be used as a ruler to quantify its mobility. Furthermore, in the fluidic device, a long-distance migration of zebrafish is eliminated in the confined space of the vibrating droplet. A high-resolution fluorescence imaging can, therefore, be performed on zebrafish, which is maintained in dynamic environmental conditions.

## Data availability statement

The original contributions presented in the study are included in the article/Supplementary material, further inquiries can be directed to the corresponding authors.

## Ethics statement

The animal study was reviewed and approved by Experimental Animal Management and Ethics Committee of Northwest University NWU-AWC-20190617Z.

## Author contributions

CZ and GJ designed the experiments, prepared the figures, and wrote the manuscript. XJ, YF, and WM generated critical preliminary data. YL and WZ performed data analysis. GJ, CZ, JT, and TY generated new data and prepared new figures for the revised manuscript. JT and CZ secured funding. All authors contributed to the article and approved the submitted version.
