# Supplementary material for: A fluidic platform for mobility evaluation of zebrafish with gene deficiency
Source: Front Mol Neurosci. 2023 Apr 6;16:1114928. doi: 10.3389/fnmol.2023.1114928 (PMC10117665; doi:10.3389/fnmol.2023.1114928)
Supplement: Supplementary file 1 [file Data_Sheet_1.pdf]

Supplementary Information
A Fluidic Platform for the Evaluation of Zebrafish Mobility

This PDF file includes:

Figs. S1 to S5
Caption for Video S1 to S8

1. Power analysis on zebrafish mobility in droplet

Our results demonstrate that in droplets with no flow, the three types of zebrafish (i.e., the wild-type, the disabled and recovered ones) cannot distinguish themselves from each other (Fig. S4). For example, the position of Fish01 distributes at 176 $\pm$ 106 °. To distinguish a position difference of 10 ° with a power of 0.8, it requires 1016 tests. To test the mobility of two zebrafish phenotypes, e.g., the ones with disrupted motor function as being compared to the wild-type samples, we obtain power of ~ 0.40 for Fish04 to Fish02; ~ 0.14 for Fish03 to Fish01; 0.07 for Fish05 to Fish01; and 0.07 for Fish06 to Fish02. These results indicate that in a droplet with no flow, mobility difference of zebrafish cannot be distinguished.

In contrast, in droplets with 2 recirculating flow, the position distribution of a typical wild-type zebrafish is 89 $\pm$ 9 ° (Fig. S5). To distinguish a position difference of 10 ° with a power of 0.8, it requires merely 9 tests. Disabled zebrafish show no obvious directional preference, which is reflected by position distribution of 177 $\pm$ 91 °. To get the same power, the number of tests required to detect 10 ° difference increases to 843 for disabled zebrafish and to 372 for the ones with recovered mobility. While, the power of the tests to distinguish disabled zebrafish (131 tests) from both wild-type (114 tests) and recovered samples (156 tests) is close to 1.

The behavioral difference is more obvious in droplet with 4 recirculating flow (Fig. S6). The position distribution of wide-type zebrafish is 90 $\pm$ 8 °, and 0 $\pm$ 8 ° for the disabled ones. It requires considerably less tests to distinguish 10 ° difference as compared to the droplet with 2 recirculating flow, i.e., 8 tests for wide-type and 11 tests for the immobile ones. When zebrafish recover from anesthesia, they gradually return to the position of wide-type fishes, i.e., 91 $\pm$ 30 ° (Fig. 5i). Even though the number of tests required to distinguish a position difference of 10 ° with a power of 0.8 increases to 74, the power of the tests to distinguish different zebrafish types remain close to 1.

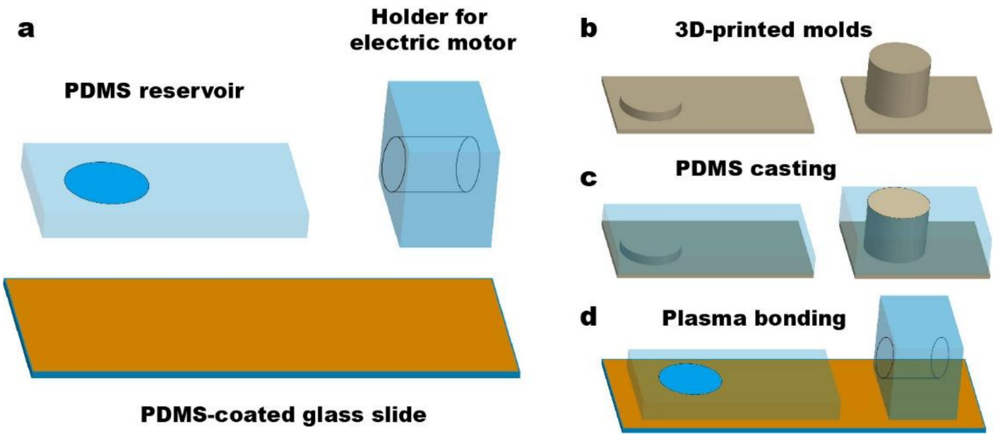

Fig. S1. a. Schematic shows the chip is composed by a glass slide spin-coated by a thin PDMS layer, a PDMS reservoir for the droplet, and a holder for the electric motor. **b.** Molds for the PDMS reservoir and holder are produced using 3D printing. **c.** PDMS is casted on 3D-printed molds and solidified. **d.** PDMS parts are bond to glass slide using plasma treatment.

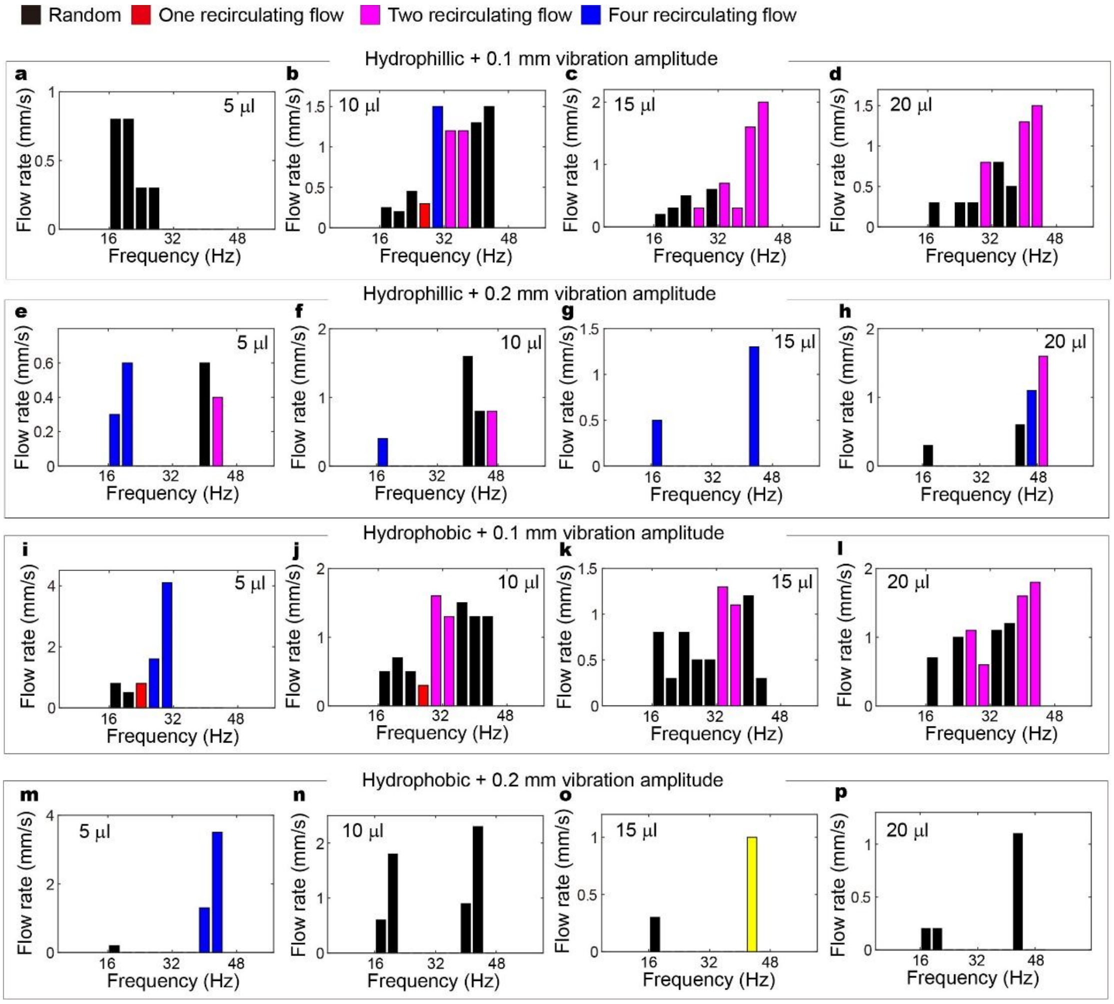

Fig. S2. Flow patterns and averaged flow rates generated in the droplet by regulating characteristics including droplet volume, shape, vibration frequency and amplitude. The flow patterns were color coded, i.e., random flow (black), one recirculating flow (red), 2 recirculating flow zones (purple), and 4 recirculating flow (blue). The average flow rate is considered as zero, when there is clear droplet deformation.

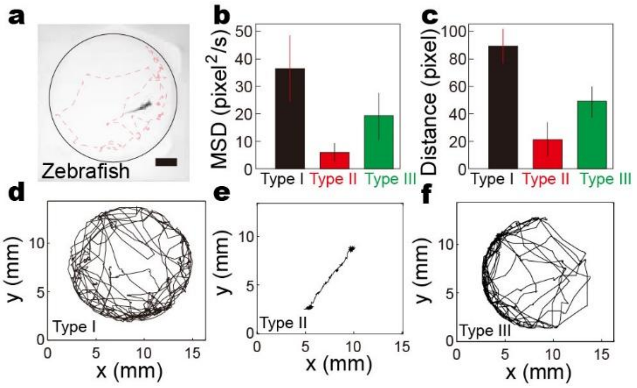

Fig. S3: Movement of different types of zebrafishes in a miniature swimming pool. (a) Snapshot of a zebrafish, which is maintained in the swimming pool, and its movement trajectory within 10 seconds. Our results demonstrate that zebrafish remain mobile without using the touch-evoked approach. (b,c) Mean square displacement (MSD) and travel distance of different zebrafishes (i.e. type I: wild type; type II: fishes with disrupted mobility; type III: recovered type II fishes) reveal that mobility of zebrafishes can be reflected by their movement. There are ~10 fishes in each group. (d-f) Trajectories of type I, type II and type III zebrafishes within the swimming pool. Scale bar denotes 2 mm.

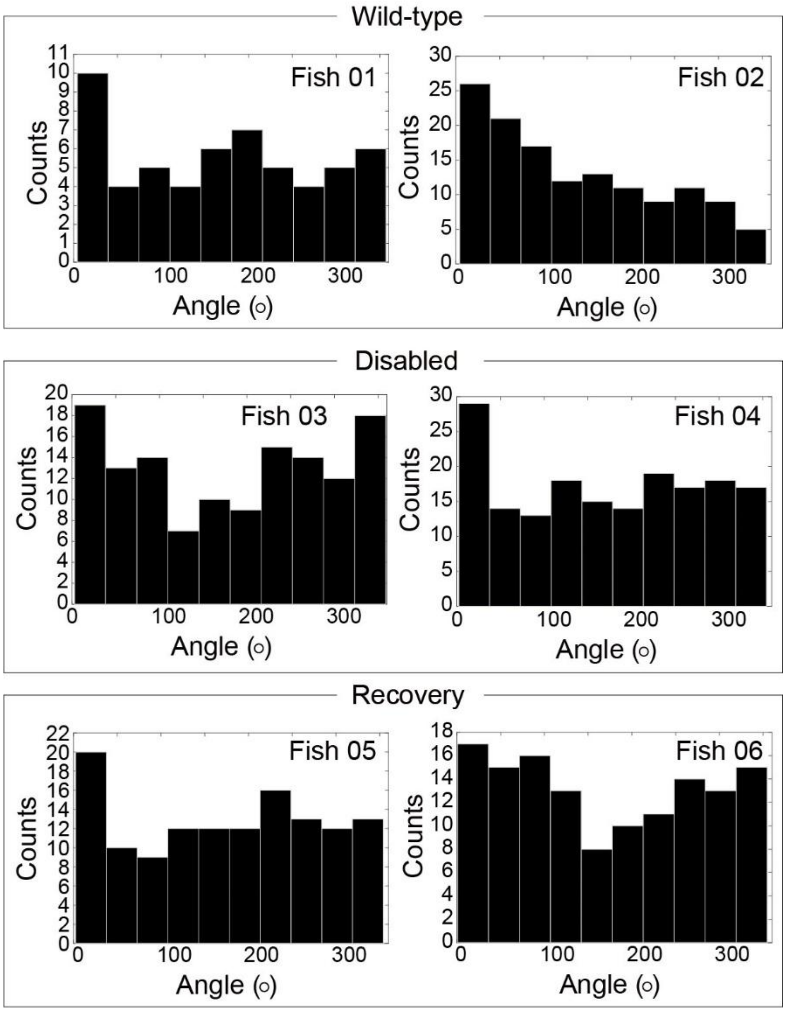

Fig. S4. Positions of different types of zebrafish in droplets with no flow, i.e., Fish01 and Fish02 are wild-type fishes; Fish03 and Fish04 are zebrafish with disrupted mobility (anesthetized); Fish05 and Fish06 are the recovered ones. It is demonstrated that different types of zebrafish show no observable differences.

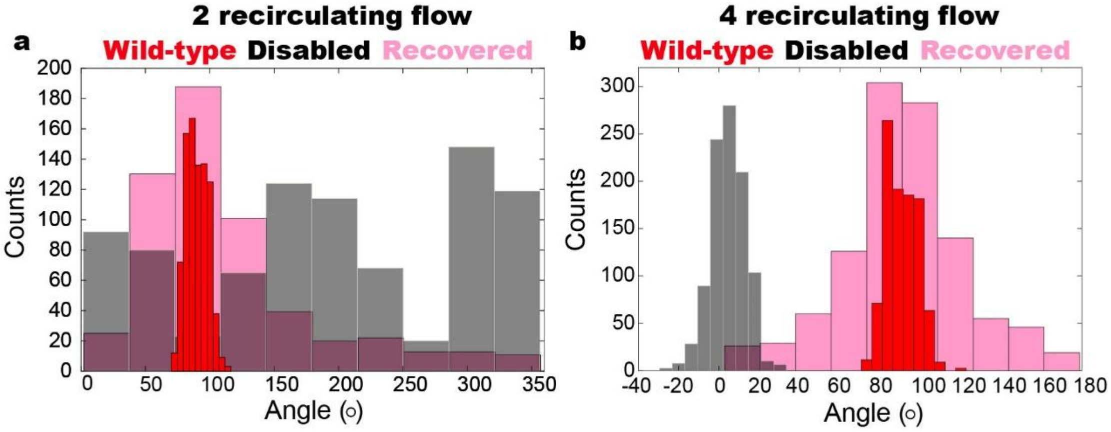

Fig. S5. Positions of different types of zebrafish in droplets with 2 and 4 recirculating flow.

Video 1: Deformation and internal flow pattern generated in a 5 ml droplet at substrate vibration amplitude of 0.2 mm and gradually changing vibration frequency. The contact angle is ~ $80°$.

Video 2: Movement of zebrafish and liquid in a 96-well plate, which was positioned on a shaker at ~ 30 Hz frequency.

Video 3: Movement of a wild-type zebrafish when being restrained in a droplet with single recirculating flow.

Video 4: Movement of a zebrafish with disrupted mobility (type II) in a droplet with 4 recirculating flow.

Video 5: Movement of wild-type zebrafish in the miniature swimming pool. The videos were recorded using a commonplace mobile camera. It is demonstrated that zebrafish may behave differently (e.g., Fish04) when no external stimulation was applied.

Video 6: Movement of a wild-type zebrafish recorded at different magnifications using Nikon Ti2E inverted fluorescence microscope.

Video 7: Scanning of a wild-type zebrafish using Nikon A1R confocal microscope.

Video 8: The device is composed by a small electric motor (M20, DC1.5V~3V), which is mounted on a glass slide and connected to 2 levers carrying a metal spring. The actuation amplitude and frequency are controlled by adjusting the spring length and electric power, respectively.
